# Recent Progress and Future Challenges in MR Electric Properties Tomography

**DOI:** 10.1155/2013/546562

**Published:** 2013-03-07

**Authors:** Ulrich Katscher, Dong-Hyun Kim, Jin Keun Seo

**Affiliations:** ^1^Philips Technologie GmbH, Research Laboratories, Roentgenstraße 24-26, 22335 Hamburg, Germany; ^2^Department of Electrical and Electronic Engineering, Yonsei University, Seoul 120-749, Republic of Korea; ^3^Department of Computational Science and Engineering, Yonsei University, Seoul 120-749, Republic of Korea

## Abstract

MR Electric Properties Tomography (EPT) is a lately developed medical imaging modality capable of visualizing both conductivity and permittivity of the patient at the Larmor frequency using *B*_1_ maps. The paper discusses the development of EPT reconstructions, EPT sequences, EPT experiments, and challenging issues of EPT.

## 1. Introduction

The knowledge of electrical tissue properties is expected to be beneficial for clinical diagnosis, therapy monitoring, and RF patient safety. Electrical tissue properties can be described by conductivity *σ* and permittivity *ϵ*, and they exhibit frequency-dependent behavior since tissues are heterogenous substances comprising insulating cell membranes and conducting electrolytes. Visualization of frequency-dependent conductivity and permittivity distribution in the range from almost dc to hundreds of MHz may expand our ability to provide diagnostic information about the physiological and pathological state of tissues and organs [1].

Due to its electromagnetic background, MRI would be a top candidate for delivering this desired knowledge of electrical tissue properties. The complex permittivity *κ* : = *ϵ* − *i*(*σ*/*ω*) at an angular frequency *ω* (assumed to be below microwave range) can be probed by the time-harmonic magnetic field **H** = (*H*_*x*_, *H*_*y*_, *H*_*z*_) through the following arrangement of time-Maxwell equations (so-called Helmholtz equation):

(1) $$\begin{array}{c} \;-\nabla^{2}H=\frac{\nabla \kappa }{\kappa }\times \left[\nabla \times H\right]+\omega^{2}\mu \kappa H, \end{array}$$

where *μ* is the magnetic permeability and *κ* is assumed to be isotropic. Here, the corresponding time-varying field is *Re*{**H** *e* ^*iωt*^}. 

At frequencies below 1 kHz, Joy et al. in 1989 [2] introduced MR current density imaging (MRCDI) which aims to provide noninvasive visualization of current density **J** = ∇×**H** inside a body by externally injecting dc current using a pair of surface electrodes and measuring the induced magnetic field **H** using MRI. In MRCDI, the induced current density **J** produces a change of the main dc magnetic field, and *H*_*z*_ is a measurable quantity by MRI since it alters the MR phase image. Hence, from MRCDI, obtaining an image of **J** = ∇×**H** requires mechanical rotation of the subject inside MRI to measure all three components of **H** [3]. In 1994 [4], MR electrical impedance tomography (MREIT) was proposed to perform the conductivity imaging at dc using the MRCDI technique. In 2001 [5], an imaging technique of MREIT without mechanical rotation, called harmonic *B*_*z*_ algorithm, was developed to provide both conductivity image and current density image. After invention of the harmonic *B*_*z*_ algorithm, MREIT has advanced rapidly [6, 7]. However, it still remains a technical problem to reduce the injection current down to a level for routine clinical use while maintaining the spatial resolution of the resulting conductivity images.

**Figure 1:**
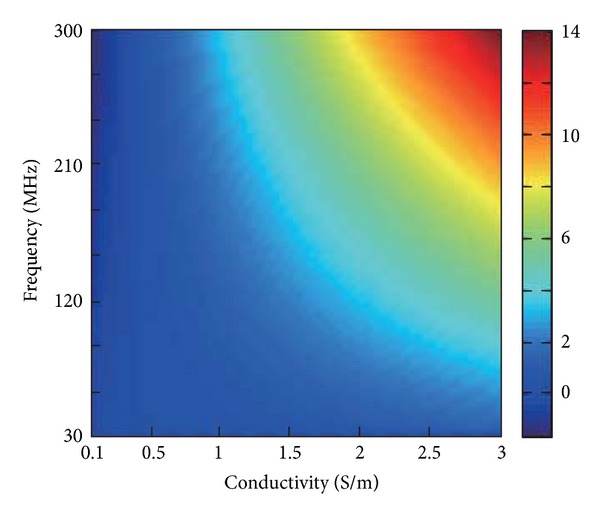
Change of magnetic RF field phase due to a brain tumor with a diameter of 1 cm. This phase change increases with the applied frequency (corresponding to the system's main field *B*_0_) as well as with the conductivity of the tumor. A mean conductivity of 0.4 S/m was assumed for the healthy parts of the brain, and thus, negative phase changes appear for assumed tumor conductivities below 0.4 S/m.

At frequencies above 1 MHz, the currents required to image electric properties have not necessarily to be injected by external surface electrodes as in MREIT. Alternatively, eddy currents can be induced by applying magnetic RF fields, avoiding the sensation of pain frequently connected with external current injection. Since magnetic RF fields are an inherent component of MRI, the desired currents can be created by standard MR systems and standard MR sequences. The resulting imaging technique, called Electric Properties Tomography (EPT) [8–11], is the subject of this paper. The basic idea of EPT is that the electric properties of the patient distort *B*_1_, the component of the magnetic RF field responsible for spin excitation. Measuring this distorted *B*_1_ by *B*_1_ mapping techniques (see, e.g., [12–17]) allows to reconstruct the electric properties causing the observed distortions. This basic idea of EPT is illustrated in Figure 1, depicting the change of the phase of the magnetic RF field due to a brain tumor with a diameter of 1 cm. This phase change increases with the applied frequency, that is, the main field *B*_0_ of the MR system used. This phase change also increases with the conductivity of the tumor. The obtained phase changes of several degrees are in a measurable range. Phase changes further increase with increasing tumor size.

RF currents induced in the tissue cause not only a distortion of *B*_1_, which is utilized for EPT as discussed above, but also off-resonance effects. As in MRCDI, these off-resonance effects can be utilized to measure the current density along the direction of *B*_0_, called RF-CDI. RF-CDI is not part of this paper, and interested readers might be referred to the corresponding literature [18–20]. The following chapters review the development of EPT reconstructions, EPT sequences, and EPT experiments.

## 2. Development of EPT Reconstruction

Without calling the approach EPT, the first mentioning of EPT was in the early nineties by Haacke et al. [8]. It was suggested to calculate both conductivity *σ* and permittivity *ϵ* via the homogeneous Helmholtz equation

(2) $$\begin{array}{c} \kappa \left(r\right)=\frac{-1}{\omega^{2}\mu_{0}}\frac{\nabla^{2}H^{+}\left(r\right)}{H^{+}\left(r\right)}, \end{array}$$

where **r** : = (*x*, *y*, *z*) and *H* ^+^ : = (*H*_*x*_ + *iH*_*y*_)/2, the positive circularly polarized component of the magnetic field corresponding to the RF transmit field. Here, the main magnetic field is $B_{0}=-B_{0}\hat{z}$, and the corresponding complex rotating vector is $a_{+}=\hat{x}-i\hat{y}$, where $\hat{x}=(1,0,0)$, $\hat{y}=(0,1,0)$,  $\hat{z}=(0,0,1)$, and *B*_0_ > 0. From (2), *σ* and *ϵ* can be expressed as

(3) σ=1ωμ0Im⁡{∇2H+H+},  ϵ=−1ω2μ0Re{∇2H+H+}.

Equation (2) is derived from (1) with the following assumptions.

(A1) A locally (“piecewise”) constant *κ*(**r**), that is; ∇*κ*(**r**) = 0. This assumption has severe consequences, which has to be discussed extensively later on.
(A2) A constant *μ*(**r**) = *μ*_0_ = 4*π* × 10^−7^, the magnetic permeability of the free space. This assumption is fairly fulfilled in the human body and does not require further discussion.
(A3) Isotropic *κ*. At the Larmor frequency of 128 MHz at 3 T, anisotropy is small in most tissues but yields an interesting niche application of EPT.
(A4) |*H* ^+^| is assumed to be larger than zero to avoid singularities in (2). This is obviously the case in areas of nonzero MR signal.

An invaluable advantage is the cancelation of the scaling of *H* ^+^ in the numerator and denominator of the expression (2). Given this cancelation, (2) yields absolute values of *κ*, even for arbitrary scaling of *H* ^+^. This feature ennobles EPT to the class of quantitative MR methods, opening the chance to directly compare *κ* between different patients and different lesions. However, in the named publication [8], EPT was not pursued further due to “*spurious phase effects unrelated to RF penetration which makes a simple extraction difficult.*” Although the mentioned spurious phase effects predominantly belonged to imperfections of the MR systems back in the early nineties, which greatly reduced since then, phase effects unrelated to RF penetration are still one of the major issues for EPT, particularly for in vivo measurements. Instead of the mentioned “*simple extraction*” of *κ*, Haacke et al. developed a heterogeneous layer model as a workaround for the observed spurious phase effects [8].

The first successful application of EPT (still not called EPT) described by Wen [9] is dated more than 10 years after Haacke's initial article. In this conference abstract, the expressions (3) are used again. Two further observations are mentioned by Wen, which later on turned out to be of central importance for EPT.

(O1) Computations of (3) require both magnitude and phase of *H* ^+^ = |*H* ^+^ | *e* ^*iϕ*^+^^. Unfortunately, only the magnitude |*H* ^+^| enters the MR signal in a nonlinear way; for each nominal flip angle *α* of the sequence, the following MR signal is measured:
  (4) $$\begin{array}{c} S\left(r\right)=V_{1}M_{0}\left(r\right)H^{-}\left(r\right)\exp\left(i\phi^{+}\left(r\right)\right)\sin\left(V_{2}\alpha \left|H^{+}\left(r\right)\right|\right), \end{array}$$
 where *M*_0_ is the MR magnitude image containing relaxation and spin density effects, *H* ^−^ = (*H*_*x*_ − *iH*_*y*_)/2, and *V*_1_, *V*_2_ system-dependent constants [21]. Thus, |*H* ^+^| has the chance to be measured exactly, assuming the ideal working of corresponding *B*_1_ mapping techniques (see, e.g., [12–17]). However, the phase *ϕ* ^+^ is difficult to be determined exactly. The phase of a standard MR image is always the superposition of *ϕ* ^+^ with its counterpart of the RF reception, *ϕ* ^−^ from *H* ^−^ and, thus, is called “transceive phase” *ϕ*_0_ = *ϕ* ^+^ + *ϕ* ^−^. In a standard MR system with a quadrature body coil (QBC), the polarization of this coil is switched from RF transmission to RF reception for the sake of optimizing SNR. Wen observed that the resulting ${\tilde{\phi }}^{-}$ of the switched QBC closely resembles *ϕ* ^+^ [9]. Consequently, a rough approximation of *ϕ* ^+^ can be obtained by the transceive phase ${\tilde{\phi }}_{0}=\phi^{+}+{\tilde{\phi }}^{-}$ of this setup
  (5) $$\begin{array}{c} \phi^{+}\approx {\tilde{\phi }}_{0}/2=\left(\phi^{+}+{\tilde{\phi }}^{-}\right)/2 \end{array}$$
 sometimes called “transceive phase assumption.”
(O2) To the leading order, the conductivity response affects the phase of the RF field, while the permittivity response affects the magnitude of the field. Thus, *σ* can be estimated by applying (2) only to *ϕ* ^+^, now called “phase-based EPT”. Accordingly, *ϵ* can be estimated by applying (2) only to |*H* ^+^|, now called “magnitude-based EPT.”

In principle, these two observations pave the way to a clinically feasible EPT. Moreover, the first successful phantom and ex vivo experiments are presented in [9], which will be topic of a later section dedicated to experimental EPT results. After this publication, Wen left the topic of EPT, and again EPT was not further pursued for years.

Systematic research on EPT started in 2009 with [10]. This publication is based on the following expression which comes from a modified Helmholtz equation:

(6) $$\begin{array}{c} \kappa \left(r\right)=\frac{\oint_{\partial A_{r}}\nabla \times H\left(r^{\prime }\right)\cdot dl}{\mu_{0}\omega^{2}\int_{A_{r}}H\left(r^{\prime }\right)\cdot dS}, \end{array}$$

where *A*_**r**_ is an arbitrarily oriented area centered at **r** with its boundary ∂*A*_**r**_, *d* **l** the line element, and *d* **S** the surface element. Equation (6) can be viewed as dividing Ampere's law by Faraday's law after suitably integrating these laws. In contrast to (2), the expression (6) requires all three components of the magnetic field **H**. Since *H* ^−^ and *H*_*z*_ are not measurable directly, *H* ^−^ = 0 and *H*_*z*_ = 0 were assumed for the applied QBC [10]. Nevertheless, a couple of basic findings studied with this modified Helmholtz equation is valid for all kinds of EPT reconstructions. First of these basic findings, the violation of assumption (A1), the locally constant *κ*, leads to severe artifacts along boundaries between compartments of different *κ*. These artifacts are typically strong oscillations (under/overshooting) of the reconstructed *κ* as shown in Figure 3. This is predominantly a question of the numerical implementation of the calculus operations of the EPT equation applied, which always involves a number of voxels in the neighborhood of the target voxel to be reconstructed. This so-called kernel of involved voxels, regardless of the actual calculus operation, causes the mentioned oscillations as soon as it contains voxels of different *κ*. Thus, as shown in [10], lowering the kernel size narrows the oscillations, and a kernel of minimal size seems to be optimal. As shown in [10], lowering the kernel size also degrades the noise figure. This touches another basic finding: the second derivative, as enclosed explicitly or implicitly in all EPT equations, tends to significantly enhance the noise in the measured *H* ^+^. For minimal kernel size, the SNR of the reconstructed *κ* is far below the SNR of the input *H* ^+^ [10, 22]. This initiated a bunch of activities to find the optimal tradeoff between artifacts and SNR or to find suitable workarounds, as discussed later.

 Besides, for the phantom used in [10], the violation of the transceive phase assumption (O1) was proven to be far below 1°, and thus errors arising from this violation are expected to be lower than errors from other sources. Last but not least, in [10], EPT was first applied to estimate local SAR.

Another milestone of EPT reconstruction is given by [11]. In contrast to the factual, “physical” modification of (2) in [10], the modification in [11] is the result of reformatting the original equation (2) by suitably integrating its numerator and denominator:

(7) $$\begin{array}{c} \kappa \left(r\right)=\frac{-\int_{\partial V_{r}}\nabla \left[\left|H^{+}\left(r^{\prime }\right)\right|e^{i\phi^{+}(r^{\prime })}\right]\cdot dS}{\mu_{0}\omega^{2}\int_{V_{r}}\left[\left|H^{+}\left(r^{\prime }\right)\right|e^{i\phi^{+}(r^{\prime })}\right]dV}, \end{array}$$

where *V*_**r**_ is a volume centered at **r** with its boundary ∂*V*_**r**_ and *dV* is the volume element. The denominator of (7) averages the denominator of (2) over a certain volume, while the numerator integrates the normal derivative to the surface of this volume. From a numerical point of view, the minimal kernel size of (7) is larger than the minimal kernel size of (2), looking for a better solution of the above-mentioned tradeoff between boundary artifacts and noise level. A systematic comparison of the behavior of (2) and (7) is conducted in [23]. Equation (7) can be rewritten with separate real and imaginary part

(8) κ=−1μ0ω2[(∇2|H+||H+|−|∇ϕ+|2)      +i(2∇ln⁡|H+|·∇ϕ++∇2ϕ+)].

As already indicated in [9], observation (O2), the assumption |∇^2^ *ϕ* ^+^ | ≫2|∇ln⁡|*H* ^+^ | ·∇*ϕ* ^+^| yields phase-based EPT for conductivity imaging

(9) $$\begin{array}{c} \sigma \left(r\right)\approx \frac{\nabla^{2}\phi^{+}\left(r\right)}{\mu_{0}\omega }, \end{array}$$

and the assumption |∇^2^|*H* ^+^|/|*H* ^+^|| ≫ |∇*ϕ* ^+^|^2^ yields magnitude-based EPT for permittivity imaging

(10) $$\begin{array}{c} \epsilon \left(r\right)\approx \frac{-\nabla^{2}\left|H^{+}\left(r\right)\right|}{\mu_{0}\omega^{2}\left|H^{+}\left(r\right)\right|}. \end{array}$$

Reference [11] derives analytically the related errors of these approaches and investigates systematically their feasibility. For typical *κ* of human tissue at 1.5 T or 3 T, the error introduced by the expressions (9)-(10) is only of the order of 10%. As can be seen directly from the corresponding error terms, phase-based EPT always yields too high conductivities, and magnitude-based EPT always yields too low permittivities. Thus, the discussed errors can be hidden by mapping not *σ* or *ϵ*, but |*κ*| as shown in [24].

Phase-based EPT reveals two features invaluable for clinical applications.

(F1) The linearity of the expression (9) supersedes the QBC transceive-phase assumption (5), allowing arbitrary combinations of RF transmit and receive coils. The resulting transceive phase, containing *ϕ* ^+^ and *ϕ* ^−^ from different RF fields, still yields *σ* via
  (11) σ=∇2ϕ0/2μ0ω=∇2(ϕ++ϕ−)2μ0ω=12(∇2ϕ+μ0ω+∇2ϕ−μ0ω)=2σ2
 since (9) can be based on *ϕ* ^+^,  *ϕ* ^−^, or any phase fulfilling Maxwell's equations, leading to the same *σ* as long as ∇|*H* ^+^ | = 0 and ∇|*H* ^−^ | = 0 are fulfilled.
(F2) Skipping the need of mapping (the magnitude of) *B*_1_, which is typically a rather lengthy scan, significantly speeds up the scan time required for EPT. As discussed below, even real-time conductivity measurements seem to be possible [25]. Moreover, it opens the chance that the conductivity can be obtained via sequences which are not primarily driven for EPT, just reusing the transceive phase which usually comes for free with every MR sequence.

Shortly after [11], which was performed at *B*_0_ = 1.5 T, phase-based EPT has been confirmed at *B*_0_ = 7 T [26]. The impact of *B*_0_ on EPT and the related question of *B*_0_ optimal for EPT turned out to be a nontrivial task [27]. Obviously, higher SNR can be expected with increasing *B*_0_. This advantage is counterbalanced by the increasing violation of the assumption ∇|*H* ^+^ | = 0 for (9) or the QBC transceive phase assumption (5), respectively. Although not explicitly stated by the authors of [27], the optimal tradeoff between SNR and reconstruction accuracy seems to be given at *B*_0_ = 3 T for conductivity imaging. For permittivity imaging, the violation of the assumption ∇*ϕ* ^+^ = 0 does not increase with *B*_0_, and the highest available *B*_0_ seems to be optimal. This trend is further emphasized by the different powers of *ω* in (3).

Since 2009, research on EPT spread out, and more and more groups started to investigate different aspects of EPT [26–31]. Typically, the original expression (2) has been used in these studies.

The problem of separating *ϕ* ^+^ and *ϕ* ^−^ from the transceive phase has been solved analytically by an approach sometimes called “Local Maxwell Tomography” (LMT) [32–34]. LMT is based on the insight that the reconstructed *κ* must not depend on the applied RF coil [31]. This is a particularly useful insight given a system with multiple, independent RF transmit channels (see, e.g., [35, 36]). Such multitransmit systems, designed primarily for RF shimming at high *B*_0_, offer the chance to determine *κ* separately by each single TX channel or any arbitrary combination of TX channels. Differences of the reconstructed *κ* based on different RF excitations can be related to a violated transceive phase assumption. For instance, two EPT reconstructions *σ*_*n*_ and *σ*_*m*_ can be compared based on different TX channels *n* and *m*, but same receive channel with phase *ϕ* ^−^, yielding [34]

(12) $$\begin{array}{c} \sigma_{n}\left(r\right)-\sigma_{m}\left(r\right)=\frac{1}{\mu_{0}\omega }\nabla \phi^{-}\left(r\right)\cdot \nabla \ln\frac{\left|H_{n}^{+}\left(r\right)\right|}{\left|H_{m}^{+}\left(r\right)\right|}. \end{array}$$

This allows, first, the determination of the unknown *ϕ* ^−^ and, subsequently, the straightforward determination of the unknown *ϕ*_*n*_^+^ and *ϕ*_*m*_^+^. The central idea of comparing reconstruction results of two or more different TX channels can also be utilized to exactly distinguish |*H* ^−^| from the spin magnetization *M*_0_, another hitherto unexplored possibility [33]. Unfortunately, the numerical effort to solve the related equations is high. It shall be clarified in future studies, if the accuracy of the obtained *ϕ* ^+^ is high enough to improve reconstruction results hitherto obtained in the framework of the discussed phase assumptions. The explicit knowledge of *ϕ* ^+^,  *ϕ* ^−^, and |*H* ^−^| seems to be more urgent for the determination of local SAR [34].

Thus, at least on a theoretical basis, the issue of phase determination is figured out, and the last remaining issue for the EPT reconstruction is the treatment of nonconstant *κ* (see Figure 3). Equation (2) can be viewed as a simplified version of (1) using the assumption (A1) of ∇*κ* = 0 so that the effect of (∇*κ*/*κ*) × [∇×**H**] in (1) is neglected. Here, *κ* might change continuously or discontinuously across boundaries of compartments with different values of *κ*. Both types of changing *κ* and the related errors have been analyzed thoroughly in [37]. In (1), the occurring partial derivatives of *κ* act as additional unknowns. It was suggested that these additional unknowns can be solved using the described comparison of different RF excitations [31]. Comparing (simulations of) two different RF excitations and therefore calling the algorithm “dual excitation algorithm,” the typical boundary artifacts were significantly reduced. Alternatively, it has been proposed to multiply (1) with ∇×**H** yielding [38]

(13) $$\begin{array}{c} \kappa \left(r\right)=-\frac{\nabla^{2}H\left(r\right)\cdot \left(\nabla \times H\left(r\right)\right)}{\omega^{2}\mu H\left(r\right)\cdot \left(\nabla \times H\left(r\right)\right)}. \end{array}$$

This equation has the big advantage of removing the term involving ∇*κ* in (1). However, computation of (13) requires all spatial components of **H** and |**H** · ∇×**H** | >0. The authors of [31, 38] propose to assume *H* ^−^ = 0 and *H*_*z*_ = 0 as it was done in connection with (6) [10]. A challenging problem is to find an exact relation between *κ* and *H* ^+^ from the full equation (1) in such a way that *κ* can be computed robustly and efficiently using *H* ^+^ only.

From a numerical point of view, the occurring oscillations along compartment boundaries are a question of finite kernel size as explained above. Thus, instead of solving (1), boundary artifacts can be avoided by image segmentation prior to reconstruction and performing separate reconstructions on the different compartments. This was demonstrated in [39] in the framework of breast EPT, where different conductivities of highly nested fatty and ductile tissue spoil a standard EPT reconstruction completely. The image segmentation can be used for shaping the applied kernel locally to the current tissue type, as well as for locally restricting a subsequently applied smoothing filter [39]. The mentioned image segmentation can be based on standard T1/T2 weighted images, which implies that same T1/T2 coincides with same *σ*. This of course is not automatically fulfilled; however, violations of this assumption are expected to occur much less than discontinuous *σ* across T1/T2 boundaries.

This chapter ends with a brief discussion of anisotropic **κ** violating (A3). Anisotropic **κ** (a rank-2 tensor) can be characterized by its eigenvectors **v**_1_, **v**_2_, and **v**_3_ (unit vectors) and its corresponding eigenvalues *κ*_1_, *κ*_2_, and *κ*_3_, respectively:

(14) $$\begin{array}{c} \kappa =\left(\begin{array}{ccc} v_{1} & v_{2} & v_{3} \end{array}\right)\left(\begin{array}{ccc} \kappa_{1} & 0 & 0\\ 0 & \kappa_{2} & 0\\ 0 & 0 & \kappa_{3} \end{array}\right)\left(\begin{array}{c} v_{1}\\ v_{2}\\ v_{3} \end{array}\right). \end{array}$$

Measuring anisotropy of the tissue conductivity, characterizing the underlying cell structure, might increase diagnostic information. In vivo, anisotropic conductivities can be found in tissue with preferred cell direction, for example, in muscles and nerves. However, one has to keep in mind that anisotropy of **κ** is expected to decrease with increasing *ω*, and anisotropy at Larmor frequency could be negligible. Nevertheless, it was pointed out that varying the orientation of the integration area *A* in (6) reflects the degree of anisotropy [40, 41]. To be precise, let *A*(**n**) be a disk with its unit normal vector **n**. Some anisotropic structure of **κ** can be observed by displaying the following quantity on the sphere *S* ^2^ [41]:

(15) $$\begin{array}{c} \Theta \left(n\right):=\frac{\oint_{\partial A(n)}\nabla \times H\cdot dl}{\mu_{0}\omega^{2}\int_{A(n)}H\cdot dS},\;n\in S^{2}. \end{array}$$

A perfectly isotropic **κ** should not depend on the direction **n** of *A*(**n**). On the other hand, the reconstructed **κ** should show a minimum for *A*(**n**) perpendicular to the (main) direction of a (strongly) anisotropic **κ**. Experimental results of a straw phantom confirmed this concept [40]. However, according to corresponding simulations [41], the concept shall not work without proper knowledge of *H* ^−^. 

## 3. EPT Sequence

### 3.1. Measuring *B*_1_ Magnitude

 As seen in (4), the transmit magnitude |*H* ^+^|, required for EPT, can be measured in a straightforward manner due to its nonlinear impact on the MR signal. In this framework, numerous techniques for |*H* ^+^| mapping (*B*_1_ mapping) are published (see, e.g., [12–17]). In principle, EPT can be based on any *B*_1_ mapping method. The accuracy of EPT depends on the accuracy of this mapping; that is, the most accurate |*H* ^+^| mapping method leads to the most accurate EPT results. Studies looking for the optimum *B*_1_ mapping technique, independent of EPT, have been published elsewhere. 

### 3.2. Measuring *B*_1_ Phase

 As discussed in the previous chapter, determination of the *B*_1_ phase *ϕ* ^+^ always starts with the measurement of the transceive phase. One of the main issues of EPT, as already pointed out in [8], is the contamination of the transceive phase by unwanted phase contributions unrelated to RF penetration. The following steps from (S1) to (S4) describes how to get a transceive phase usable for EPT reconstruction.

(S1) The transceive must not contain any contributions from *B*_0_, that is, any off-resonance effects. The easiest way to exclude off-resonance effects is the use of refocusing pulses, that is, sequences based on spin echoes (SE), like fast spin echo sequences, turbo spin echo sequences, and so on. In contrast, the transceive phase of field-echo based sequences includes off-resonance effects. In this case, these effects can be removed by any kind of *B*_0_ mapping. In the easiest way, the phase can be measured at two different TE and extrapolated back to TE = 0 [26]. Also more sophisticated *B*_0_ maps can be applied, for example, obtained in the framework of the Dixon techniques (see, e.g., [42]). On the other hand, sequences with balanced gradients (steady-state free-precession, SSFP) are known to have benign off-resonance behavior [25]. As long as *B*_0_ inhomogeneities are too small to cause the well-known banding artifacts, which lead to phase jumps of 180°, the SSFP transceive phase fairly resembles the transceive phase of spin echo sequences [25].
(S2) The transceive phase must not contain any contributions from eddy currents in the tissue induced by gradient switching. This can be obtained by averaging two separate measurements with inverted gradient polarization [43]. Alternatively, the balanced gradients of SSFP sequences eliminate this unwanted phase contribution automatically [25].
(S3) Phase contributions from flow and motion should be removed or at least suppressed as much as possible. This job can be done, for example, by double spin echo sequences [44] or, again, by SSFP [25].
(S4) If the “full” complex EPT equation has to be solved and not the phase-based version of EPT, the transceive phase has to be unwrapped before being divided by two, (5). This unwrapping in the three spatial dimensions can be facilitated by performing it separately for each differentiation.

Thus, if applicable, SSFP sequences seem to be the sequence of choice for EPT transceive phase measurements. Due to its high efficiency, SSFP is also a good candidate for real-time phase-based EPT [25] as discussed below. 

### 3.3. RF Shimming

 More and more MR systems at *B*_0_ = 3 T and above are equipped with multiple, independent RF transmit channels (see, e.g., [35, 36]). The primary goal of this technique is the compensation of *B*_1_ inhomogeneities via RF shimming, that is, the patient-individual adjustment of the complex TX channel weights. It is important to recognize that *B*_1_ fields obtained by this RF shimming still satisfy the Helmholtz equations (1) and (2). Thus, if |*H* ^+^| and *ϕ* ^+^ of the shimmed system are measured correctly, the accuracy of EPT is not affected. However, the following topics have to be kept in mind regarding EPT in combination with RF shimming.

- RF shimming allows RF excitation far from quadrature excitation. Thus, one has to be aware that the transceive phase assumption (5), derived for a QBC with switched polarization, can be significantly violated [45].
- Phase-based EPT assumes |*H* ^+^| = const, which can be supported by RF shimming [46]. Obviously, RF shimming impairs magnitude-based EPT. Magnitude-based EPT would benefit from RF phase shimming, as long as the resulting *B*_1_ inhomogeneities do not cause signal voids, violating assumption (A4).
- Having the complex *B*_1_ maps of all TX channels at hand, RF shimming can be performed a posteriori, adjusting the channel weights sequentially for certain ROIs or even voxelwise. Thus, the resulting total *B*_1_ can be optimized locally prior to local EPT reconstructions, as was pointed out by [33].

### 3.4. Hybrid Sequences

 Since phase-based EPT in principle just requires a standard image's transceive phase, it is more or less straightforward to combine phase-based EPT with all kinds of other sequences. Below, two possible combinations are depicted. A lot more are expected to come up in the near future. One fruitful combination is obtained by performing EPT and MREIT simultaneously [37]. By injecting external current in the patient for a few ms between RF pulse and signal sampling, MREIT is able to extract tissue conductivity at frequencies below 1 kHz corresponding to the duration of the current injection. Conductivity determined with EPT is related to the Larmor frequency. Thus, the EPT/MREIT hybrid sequence yields a minimal form of conductivity “spectrum.” This frequency dependence can be strikingly proved by wrapping (parts of) phantoms in insulating wrap. The EPT result is not at all affected by the wrapping. For MREIT, the apparent conductivity of the wrapped part is completely suppressed [37]. We refer to [47] that explains how high-frequency current can penetrate the thin membrane and also how this is linked to the frequency-dependent behavior of the complex potential.

Another hybrid sequence is described in [48], where EPT is combined with quantitative mapping of the susceptibility *χ* (QSM). This combination is driven by the idea to get a complete electromagnetic description of the tissue. EPT and QSM are based on two superposed components of the phase of a gradient echo image. QSM requires the phase component related to *B*_0_ and off-resonance effects; EPT requires the phase component related to *B*_1_, that is, RF transmission and reception as described in Section 3.2. The separation of these two components, for example, by extrapolating the phase of a multiecho sequence to TE = 0 as described above, is the basis of both EPT and QSM. Interested only in EPT or QSM, the noninteresting phase component is just dropped. Thus, the combination of EPT and QSM is simply done by using both phase components as inputs for the respective approaches [48].

## 4. EPT Experiments

### 4.1. Phantom Studies

 The principle feasibility of EPT has first been proven using phantoms with different saline concentrations covering roughly the physiologic range, showing a correlation of more than 99% between expected and obtained conductivities [9, 10]. A corresponding permittivity study was published in [49]. For low *B*_0_, noise in permittivity images is tremendously higher than in conductivity images, reflecting the low bending of |*H* ^+^| for *B*_0_ < 3T. For increasing *B*_0_, the SNR gain is higher for permittivity than for conductivity, which arises from different powers of *ω* in (3) [24, 49].

The possibility of fast phase-based conductivity imaging has been tested with adding NaCl to a tap water phantom during scanning [25]. The applied SSFP sequence measured images of the whole phantom (size 1 liter, resolution 2.5 × 2.5 × 2.5 mm^3^) within 4 seconds, visualizing the formation of laminar layers with different salt contents and conductivities at the bottom of the phantom.

Conductivity increases with temperature by roughly 2% per Kelvin. This feature has been confirmed with EPT by heating/cooling different phantoms with water and biologic substances (muscle sample, tomato and apple puree) [50]. For the obtained measurement accuracy, no difference in the conductivity's temperature behavior between the different phantoms has been found. This approach might be helpful in the framework of hyperthermia and thermoablative interventions.

### 4.2. In Vivo Studies: Brain Applications

 First EPT in vivo studies have been reported by Voigt et al., investigating different volunteers' brains [11]. His results reveal several central features of in vivo EPT.

- The conductivity and permittivity of healthy grey matter, white matter, and cerebrospinal fluid (CSF) measured with EPT coincide with the literature values [51].
- As expected from theory, phase-based EPT decreases obtained conductivity values by 10%, and magnitude-based EPT increases obtained permittivity values by 10% (see Table 1).
- Comparing different volunteers, results vary by roughly 15% for gray and white matter and 20% for CSF. These data comprise measurement uncertainties as well as physiologic intersubject differences.

The intersubject variability can be compared with the intra-subject variability, which has been investigated in [52]. Scanning a single volunteer 10 times over a period of three weeks, conductivity variations of roughly 10% across the gray/white matter was obtained. Since this value is close to the intersubject variability reported in [11], physiologic differences between volunteers appear to be rather small. On the other hand, intra-object conductivity variations of phantoms are far below 1%, making system instabilities an unlikely reason for the observed intra-subject variability of 10%. Alternative reasons could be subject motion, spontaneous conductivity fluctuations caused by nutrition or state of health, or others, yet unidentified subject-induced phase instabilities (“unrelated to RF penetration,” see [8]).

**Figure 2:**
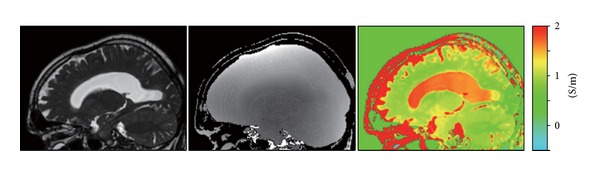
Volunteer brain study. Left: SSFP image (magnitude); center: SSFP image (phase); right: reconstructed conductivity based on the Laplacian of the SSFP phase and locally restricting the subsequent filtering using the SSFP magnitude.

**Figure 3:**
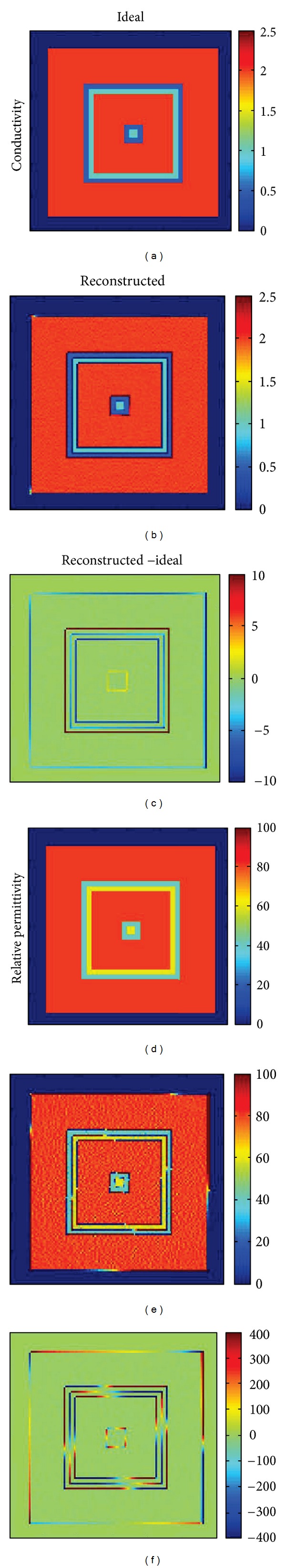
Local inhomogeneity effects using the Helmholtz equation. The reconstructed conductivity and permittivity show strong oscillations. (Simulations were done for a cylinder object of size 100 mm × 100 mm × 120 mm with 3 different values of conductivity (2 S/m, 1 S/m, and 0.5 S/m) and relative permittivity (80, 60, and 40).)

The conductivity map of a brain of a healthy volunteer is shown in Figure 2. EPT is based on the transceive phase of an SSFP scan using a head coil at 1.5 T and a scan time of 3 minutes for an isotropic resolution of 1 × 1 × 1 mm^3^. Boundary artifacts were reduced with a median filter, locally restricted by the SSFP magnitude image.

For most clinical EPT studies, the focus of interest is on oncology, particularly brain and breast tumors. Initial, single cases of brain tumors have been reported for 1.5 T [53] and 7 T [54]. All cases show a tumor conductivity increased roughly by a factor of two compared with the surrounding white matter, as expected from [55]. In [54], the hypothesis is raised that the tumor conductivity correlates with its sodium content. Systematic studies on a larger number of brain tumor patients, trying to understand the biochemical reasons for enhanced tumor conductivity and to classify different kinds of tumors, are on the way. A single stroke patient was described by [56]. Again, a clear increase of the conductivity within the stroke area has been observed.

### 4.3. In Vivo Studies: Body Applications

#### 4.3.1. Breast

**Figure 4:**
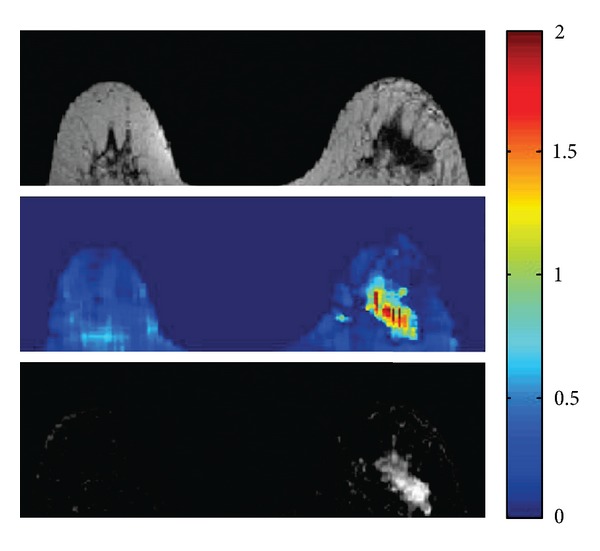
Breast tumor patient study. Top: turbo spin echo (magnitude); center: reconstructed conductivity image; bottom: contrast enhanced dynamic image. The dynamic image shows the region of the tumor.

The main components of breast tissue, gland and fat, differ significantly in *σ*. These components are typically highly nested, leading to high amount of significant conductivity discontinuities throughout the breast. These discontinuities are able to completely spoil any standard EPT reconstruction based on (2) or (9) and (10). Instead of handling conductivity discontinuities on a physical basis via (1), boundary artifacts can be avoided by image segmentation prior to reconstruction and performing separate reconstructions on the different compartments as demonstrated in [39]. Here, the image segmentation was used for shaping the applied kernel locally to the current tissue type, as well as for locally restricting the subsequently applied median filter. The image segmentation was based on the magnitude image of the 3D turbo spin echo performed to obtain the transceive phase for the EPT reconstruction. The resulting conductivity map is more or less free of boundary artifacts. The tumor clearly shows the highest conductivity throughout the breast *σ* ~2.0–2.5 S/m. Several cysts show an intermediate conductivity *σ* ~1.0–1.5 S/m. The conductivity of the surrounding fatty tissue is around zero *σ* ~±0.5 S/m. Remaining inhomogeneities particularly in the fat compartments might arise from insufficient *B*_1_ homogeneity or, more likely, from tissue eddy currents, since no second scan with inverted gradient polarization has been performed ([43], step (S2) in the above list).  Figure 4 shows breast tumor patient study. Further breast studies are on the way (see, e.g., the breast permittivity study [57]).

#### 4.3.2. Heart

Two isolated, perfused pig hearts where scanned with a gated SSFP sequence [58]. Conductivity values from phase-based EPT of normally perfused heart tissue were compared with the values in ischemic regions after a blockade of the left anterior descending artery. Normal conductivity values turned out to be in agreement with the literature values, while conductivity in ischemic/infarcted areas was 60% lower than that in remote myocardium [58]. These findings are very encouraging for future, challenging in vivo cardiac experiments.

#### 4.3.3. Liver

By analogy to the above-described brain studies, the intra-subject variability of the liver conductivity of healthy volunteers has been investigated [59]. The high efficiency of the applied SSFP sequence allows scanning the whole liver within a single breathhold. The obtained intra-subject and intersubject variability was of the same range as for the brain [11, 52]. A higher artifact level was observed in the liver than in the brain, presumably arising from cardiac motion transferred to the liver and only incompletely suppressed by the applied SSFP sequence. Artifacts are more pronounced for expiration than inspiration breathhold, which can be explained by the contact of heart and liver closer in expiration than in inspiration [59].

#### 4.3.4. Pelvis

In preparation of future pelvis tumor studies, the applicability of phase-based EPT was checked with a pelvis-sized phantom at 3 T [60]. Due to the larger dimensions of the pelvis, as compared to the head, the phase error has to be reinvestigated for this particular anatomy. According to this study, phase-based EPT seems to be sufficient when focusing only on the tumors; however, the inclusion of |*H* ^+^| could be necessary, when *σ* of the whole pelvis is required [60].

## 5. Conclusion

Tomographic imaging of the conductivity and permittivity distributions inside the human body has been an active research topic in the field of Electrical Impedance Tomography (EIT) measuring boundary current-voltage data [61]. However, wide experience in EIT for more than three decades has shown its methodological limitation in achieving robust reconstructions of static conductivity and permittivity images; EIT is insensitive to any perturbation of internal admittivity whereas it is very sensitive to forward modeling errors. It seems that boundary measurements are insufficient for robust reconstruction of the admittivity distribution inside the subject. For a robust reconstruction, we need an internal measurement that is capable of by MRI scanners as discussed in this paper.

Using standard MR systems and standard MR sequences, mapping of the electric properties seems to be clinically feasible, particularly phase-based conductivity imaging. The rapidly evolving field will certainly afford further improved measurement and reconstruction techniques in the near future. The broad spectrum of started preclinical and clinical studies raise hope that answers will soon be available concerning potential diagnostic benefits of EPT.

## Figures and Tables

**Table 1:** Electric properties of different compartments of the brain of a healthy volunteer, measured with EPT at 1.5T [11]. The measured values agree with values expected from the literature [51]. Phase-based EPT yields slightly increased conductivities; magnitude-based EPT yields slightly decreased permittivities.

	Conductivity [S/m]			Relative permittivity		
	Full EPT	Phase-based	Literature	Full EPT	Magnitude-based	Literature
	(7)	EPT (9)	[51]	(7)	EPT (10)	[51]
Gray matter	0.69 ± 0.14	0.72 ± 0.15	0.51	103 ± 69	91 ± 70	97.4
White matter	0.39 ± 0.15	0.43 ± 0.15	0.29	72 ± 64	63 ± 66	67.8
Cerebrospinal fluid	1.75 ± 0.34	1.82 ± 0.37	2.07	104 ± 21	98 ± 20	97.3

## References

[1] Grimnes S, Martinsen OG (2000). *Bioimpedance and Bioelectricity Basics*.

[2] Joy ML, Scott GC, Henkelman RM (1989). In vivo detection of applied electric currents by magnetic resonance imaging. *Magnetic Resonance Imaging*.

[3] Scott GC, Joy MLG, Armstrong RL, Henkelman RM (1991). Measurement of nonuniform current density by magnetic resonance. *IEEE Transactions on Medical Imaging*.

[4] Woo EJ, Lee SY, Mun CW Impedance tomography using internal current density distribution measured by nuclear magnetic resonance.

[5] Seo JK, Yoon JR, Woo EJ, Kwon O (2003). Reconstruction of conductivity and current density images using only one component of magnetic field measurements. *IEEE Transactions on Biomedical Engineering*.

[6] Woo EJ, Seo JK (2008). Magnetic resonance electrical impedance tomography (MREIT) for high-resolution conductivity imaging. *Physiological Measurement*.

[7] Seo JK, Woo EJ (2011). Magnetic resonance electrical impedance tomography (MREIT). *SIAM Review*.

[8] Haacke EM, Pepropoulos LS, Nilges EW, Wu DH (1991). Extraction of conductivity and permittivity using magnetic resonance imaging. *Physics in Medicine and Biology*.

[9] Wen H Non-invasive quantitative mapping of conductivity and dielectric distributions using the RF wave propagation effects in high field MRI.

[10] Katscher U, Voigt T, Findeklee C, Vernickel P, Nehrke K, Dossel O (2009). Determination of electrical conductivity and local SAR via *B*
_1_ mapping. *IEEE Transactions on Medical Imaging*.

[11] Voigt T, Katscher U, Doessel O (2011). Quantitative conductivity and permittivity imaging of the human brain using electric properties tomography. *Magnetic Resonance in Medicine*.

[12] Akoka S, Franconi F, Seguin F, Le Pape A (1993). Radiofrequency map of an NMR coil by imaging. *Magnetic Resonance Imaging*.

[13] Barker GJ, Simmons A, Arridge SR, Tofts PS (1998). A simple method for investigating the effects of non-uniformity of radiofrequency transmission and radiofrequency reception in MRI. *British Journal of Radiology*.

[14] Stollberger R, Wach P (1996). Imaging of the active *B*
_1_ field in vivo. *Magnetic Resonance in Medicine*.

[15] Yarnykh VL (2007). Actual flip-angle imaging in the pulsed steady state: a method for rapid three-dimensional mapping of the transmitted radiofrequency field. *Magnetic Resonance in Medicine*.

[16] Sacolick LI, Wiesinger F, Hancu I, Vogel MW (2010). *B*
_1_ mapping by Bloch-Siegert shift. *Magnetic Resonance in Medicine*.

[17] Nehrke K, Börnert P (2012). DREAM-a novel approach for robust, ultrafast, multislice *B*
_1_ mapping. *Magnetic Resonance in Medicine*.

[18] Scott GC, Joy MLG, Armstrong RL, Henkelman RM (1992). RF current density imaging in homogeneous media. *Magnetic Resonance in Medicine*.

[19] Scott GC, Joy MLG, Armstrong RL, Henkelman RM (1995). Rotating frame RF current density imaging. *Magnetic Resonance in Medicine*.

[20] Wang D, DeMonte TP, Ma W, Joy MLG, Nachman AI (2009). Multislice radio-frequency current density imaging. *IEEE Transactions on Medical Imaging*.

[21] Hoult DI (2000). The principle of reciprocity in signal strength calculations—a mathematical guide. *Concepts in Magnetic Resonance*.

[22] Shin J, Lee J, Seo JK, Kim DH Quantification error in MREPT due to *B*
_1_ map inaccuracy.

[23] Bulumulla SB, Lee SK, Yeo TBD Calculation of electrical properties from *B*
_1_
^+^ maps—a comparison of methods.

[24] van Lier ALHMW, Katscher U, Raaijmakers A, van den Berg CAT Wave-number imaging at 7T: increasing accuracy of EPT at high field strengths.

[25] Stehning C, Voigt TR, Katscher U Real-Time conductivity mapping using balanced SSFP and phase-based reconstruction.

[26] van Lier AL, Brunner DO, Pruessmann KP (2012). *B*
_1_
^+^ phase mapping at 7T and its application for in vivo electrical conductivity mapping. *Magnetic Resonance in Medicine*.

[27] van Lier AL, Raaijmakers A, Voigt T, Lagendijk JJ, Katscher U, van den Berg CA (2013). Electric properties tomography in the human brain at 1.5, 3, and 7 T: a comparison study. *Magnetic Resonance in Medicine*.

[28] Bulumulla SB, Yeo TB, Zhu Y Y Direct calculation of tissue electrical parameters from *B*
_1_ maps.

[29] Cloos MA, Bonmassar G Towards direct *B*
_1_ based local SAR estimation.

[30] Buchenau S, Haas M, Hennig J, Zaitsev M A comparison of local SAR using individual patient data and a patient template.

[31] Zhang X, Zhu S, He B (2010). Imaging electric properties of biological tissues by RF field mapping in MRI. *IEEE Transactions on Medical Imaging*.

[32] Katscher U, Findeklee C, Voigt T Single element SAR measurements in a multi-transmit system.

[33] Sodickson DK, Alon L, Deniz CM Local Maxwell tomography using transmit-receive coil arrays for contact-free mapping of tissue electrical properties and determination of absolute RF phase.

[34] Katscher U, Findeklee C, Voigt T (2012). *B*
_1_-based specific energy absorption rate determination for nonquadrature radiofrequency excitation. *Magnetic Resonance in Medicine*.

[35] Setsompop K, Wald LL, Alagappan V (2006). Parallel RF transmission with eight channels at 3 Tesla. *Magnetic Resonance in Medicine*.

[36] Vernickel P, Röschmann P, Findeklee C (2007). Eight-channel transmit/receive body MRI coil at 3T. *Magnetic Resonance in Medicine*.

[37] Seo JK, Kim MO, Lee J (2012). Error analysis of nonconstant admittivity for MR-based electric property imging. *IEEE Transactions on Medical Imaging*.

[38] Nachman AI, Wang D, Ma W, Joy MLG A local formula for inhomogeneous
complex conductivity as a function of the RF magnetic field.

[39] Katscher U, Djamshidi K, Voigt T Estimation of breast tumor conductivity using parabolic phase fitting.

[40] Katscher U, Voigt T, Findeklee C Estimation of the anisotropy of electric conductivity via *B*
_1_ mapping.

[41] Lee J, Song Y, Choi N, Cho S, Seo JK, Kim DH Noninvasive measurement of conductivity anisotropy at larmor frequency using MRI.

[42] Glover GH, Schneider E (1991). Three-point Dixon technique for true water/fat decomposition with B0 inhomogeneity correction. *Magnetic Resonance in Medicine*.

[43] Bernstein MA, King KF, Zhou XJ (2004). *Handbook of MRI Pulse Sequences*.

[44] Choi N, Ghim M, Yang S, Zho S-Y, Kim D-H In vivo conductivity mapping using double spin echo for flow effect removal.

[45] Katscher U, Findeklee C, Voigt T Experimental estimation of local SAR in a multi-transmit system.

[46] Katscher U, van Lier A, van den Berg C, Keupp J RF Shimming improves phase-based conductivity imaging.

[47] Kim S, Lee EJ, Woo EJ, Seo JK (2012). Asymptotic analysis of the membrane structure to sensitivity of frequency-difference electrical impedance tomography. *Inverse Problems*.

[48] Kim DH, Gho SM, Choi N, Liu C Simultaneous electromagnetic property imaging using multiecho gradient echo.

[49] Katscher U, Karkowski P, Findeklee C, Voigt T Permittivity determination via phantom and in vivo *B*
_1_ mapping.

[50] Leussler C, Karkowski P, Katscher U Temperature dependant conductivity change using MR based electric properties tomography.

[51] Gabriel S, Lau RW, Gabriel C (1996). The dielectric properties of biological tissues—II. Measurements in the frequency range 10 Hz to 20 GHz. *Physics in Medicine and Biology*.

[52] Stehning C, Voigt TR, Katscher U (2012). Reproducibility study of 3D SSFP phase-based brain conductivity imaging. *MAGMA*.

[53] Voigt T, Vaterlein O, Stehning C, Katscher U, Fiehler J In vivo glioma characterization using MR conductivity imaging.

[54] van Lier A Electrical conductivity imaging of brain tumours.

[55] Lu Y, Li B, Xu J, Yu J (1992). Dielectric properties of human glioma and surrounding tissue. *International Journal of Hyperthermia*.

[56] van Lier A, Kolk A, Brundel M Electrical conductivity in ischemic stroke at 7.0 Tesla: a case study.

[57] Bulumulla S, Hancu I Breast permittivity imaging.

[58] Voigt T, Schuster A, Ishida M Conductivity imaging of an Ischemic pig heart model using electric properties tomography.

[59] Stehning C, Voigt T, Karkowski P, Katscher U Electric properties tomography (EPT) of the liver in a single breathhold using SSFP.

[60] Balidemaj E, van Lier AL, Nederveen AJ, Crezee J, van den Berg CAT Feasibility of EPT in the Human Pelvis at 3T.

[61] Holder D (2005). *Electrical Impedance Tomography: Methods, History and Applications*.

